# ACE2-Targeting antibody suppresses SARS-CoV-2 Omicron and Delta variants

**DOI:** 10.1038/s41392-022-00913-3

**Published:** 2022-02-09

**Authors:** Jianxia Ou, Yanan Zhang, Yongmei Wang, Zherui Zhang, Hongping Wei, Junping Yu, Qi Wang, Guifeng Wang, Bo Zhang, Chunhe Wang

**Affiliations:** 1 Biotherapeutics Discovery Research Center, Shanghai Institute of Materia Medica, Shanghai, China; 2 School of Chinese Materia Medica, Nanjing University of Chinese Medicine, Nanjing, China; 3 Key Laboratory of Special Pathogens and Biosafety, Wuhan Institute of Virology, Center for Biosafety Mega-Science, Chinese Academy of Sciences, Wuhan, China; 4 University of Chinese Academy of Sciences, Beijing, China

**Keywords:** Infection, Cell biology

**Dear Editor**,

The pandemic of COVID-19 continues worldwide with many variants arising, especially SARS-CoV-2 Delta (B.1.617.2) and Omicron (B.1.1.529) variants of concern (VOCs). According to WHO, Omicron has spread in almost all the countries with a doubling time less than Delta VOC. Equipped with increased transmissibility and decreased responses to neutralizing monoclonal antibodies (mAbs),^1–3^ Omicron’s frequency has risen from 0.8% of all sequenced SARS-CoV-2 viral samples to 98.3% in just 40 days (https://covid.cdc.gov/covid-data-tracker/#variant-proportions), faster than Delta. Effective therapies that can limit the transmissibility of SARS-CoV-2 Omicron and Delta VOCs are therefore in urgent need.

There is no mAb or small molecule therapies specifically designed to block the spreading of Omicron or Delta VOCs. Although Delta variant was reported susceptible to bamlanivimab plus etesevimab treatment in laboratory studies and also in two Delta vaccine breakthrough individuals,^4^ such treatment regimen showed no effect on SARS-CoV-2 Gamma (P.1) and Beta VOCs (B.1.351), and its distribution was paused briefly in the United States because of that (FDA). Omicron VOC was reported to escape the current neutralizing mAbs designed against the original version.^1–3^ Since the proportions of circulating VOCs evolve rapidly, it is important to develop a therapy that could potentially control the spreading of the current and future SARS-CoV-2 VOCs.

In our previous study, 3E8, a mAb targeting human angiotensin-converting enzyme 2 (hACE2), could block the S1-subunits binding to ACE2 and pseudo-typed virus infection of ACE2-expressing cells from multiple coronaviruses, including SARS-CoV-2 VOC Gamma and Beta, without markedly affecting the physiological activities of hACE2 or causing severe toxicities in hACE2 “knock-in” mice. 3E8 also blocked the live SARS-CoV-2 infection of Vero E6 cells and in a prophylactic mouse model of COVID-19.^5^ In this study, we showed that 3E8 could potentially block Delta and Omicron VOCs, demonstrating further that it was a potent and “broad-spectrum” blocker of all coronaviruses that utilize hACE2 as entry receptors.

The binding affinities of 6× His-tagged S1 proteins of wild-type (WT), Delta, Kappa (B.1.617.1) and Omicron variants of SARS-CoV-2, as well as 3E8 to Fc-tagged hACE2 were measured. The *EC*_*50*_ values by ELISA were determined to be >4, 0.497, 0.216, >33, and 0.029 μg/ml (Fig. 1a), and the apparent dissociation constant (*KD*) by biolayer interferometry (BLI) were 16.2, 5.03, 4.85, 7.40, and 1.6 nM, respectively (Fig. 1b). They could also bind to HEK293/hACE2/EGFP cells ectopically-expressing hACE2 and Vero E6 cells endogenously-expressing ACE2, as demonstrated by flow cytometry (Fig. 1c). The binding abilities of the four S1-subunits to hACE2 were ranked in the sequence of Delta > Kappa > WT > Omicron, which may underlie the epidemiologic characteristics that the Delta variant is more contagious than previous variants, except Omicron, which exhibited weaker affinity to ACE2, but greater transmissibility. 3E8 exhibited higher affinity than S1-subunits from WT, Delta, Kappa and Omicron SARS-CoV-2 to hACE2.

**Fig. 1:**
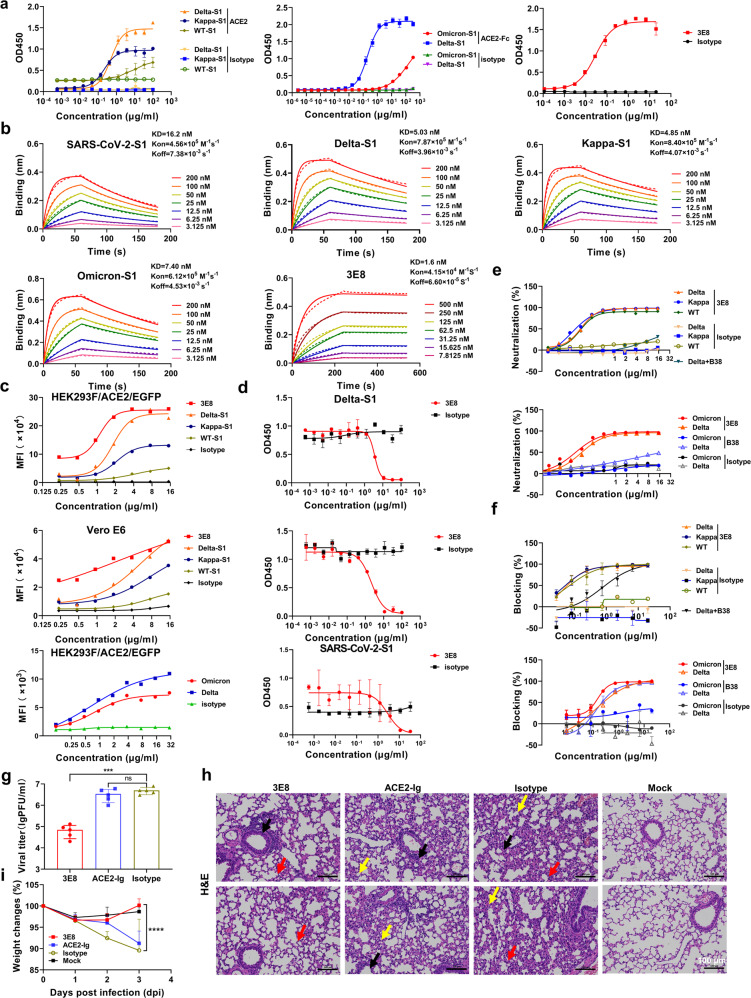
3E8 suppressed SARS-CoV-2 Omicron and Delta variants. Binding of different S1-subunits and 3E8 to hACE2 protein as measured by **a** ELISA and **b** BLI. **c** Bindings of different S1-subunits to HEK293/hACE2/EGFP and Vero E6 cells by flow cytometry. 3E8 blocked the binding of different S1-subunits to hACE2 by **d** ELISA and **e** HEK293/hACE2/EGFP cells by flow cytometry. **f** 3E8 blocked infections of HEK293/hACE2/EGFP cells by pseudo-typed coronaviruses. **g** 3E8 suppressed live Delta VOC infection in a prophylactic mouse model of COVID-19. RBD-targeting ACE2-Ig and isotype were used as positive and negative controls, respectively. **h** H&E staining of lung organ samples from different groups. ACE2-Ig- and isotype-treated mice developed serious interstitial pneumonia characterized by a large area of alveolar septal thickening (black arrow) and a large amount of inflammatory cell infiltration (red arrow) coupled with macrophage infiltration (yellow arrow). In contrast, less inflammatory cell infiltration (red arrow) was observed in the lungs of 3E8-treated mice. The scale represents 100 μm. **i** body weight changes. ***P < 0.001; ****P < 0.0001; ns, not significant

3E8 effectively blocked S1-subunits from WT, Delta and Kappa SARS-CoV-2 binding to hACE2 with comparable *IC*_*50*_ values at 2.822, 3.534 and 2.136 μg/ml (Fig. 1d), and also to HEK293/hACE2/EGFP cells ectopically-overexpressing hACE2 with comparable *IC*_*50*_ values at 0.11, 0.12, and 0.07 μg/ml, respectively (Fig. 1e). Compared to Delta VOC, Omicron showed comparable sensitivity to 3E8 (Fig. 1e). However, B38, a SARS-CoV-2 RBD-targeting antibody currently under clinical development, showed only borderline neutralization on Omicron.

Pseudo-typed coronaviruses with full-length S-proteins from WT, Delta, Kappa and Omicron SARS-CoV-2 were constructed and they could all infect HEK293/hACE2/EGFP cells ectopically-expressing hACE2. 3E8 treatment fully abolished the infectivity of WT, Delta and Kappa pseudo-viruses with similar *IC*_*50*_ values at 0.07, 0.06, 0.05, and 0.06 μg/ml, respectively. The neutralizing *IC*_*50*_ value of 3E8 on Omicron was comparable to those on previously reported variants by our group including SARS-CoV-2-D614G (0.02 μg/ml), B.1.1.7 (Alpha, 0.02 μg/ml) and B.1.351 (Beta, 0.05 μg/ml). In comparison, B38 were 10-fold weaker in suppressing Omicron (*IC*_*50*_: 1.346 μg/ml) than Delta (*IC*_*50*_: 0.137 μg/ml) (Fig. 1f). Thus, 3E8 could effectively block all the four pseudo-typed coronaviruses regardless of the mutation status of their S-proteins.

Furthermore, the neutralization of 3E8 on live Delta variant was validated in a prophylactic mouse model of COVID-19 generated in hACE2 “knock-in” mice. Consistent with our in vitro results, 3E8 could protect the mice from Delta variant infection, resulting in an ~100-fold reduction in viral loads (Fig. 1g), ameliorated the tissue damages (Fig. 1h), and protected the mice from body weight loss (Fig. 1i). In contrast, ACE2-Ig, a recombinant protein targeting the RBD protein, did not inhibit virus replication and only slightly improved the body weight loss. Due to the lack of live virus of Omicron VOC currently in the laboratory, the neutralizing effect of 3E8 on this VOC could not yet be validated in animal model.

Together with our previous study,^5^ our data suggest that 3E8 is potentially a potent and “broad-spectrum” blocker of multiple coronaviruses that utilize ACE2 as entry receptors, including SARS-CoV-2 Delta, Omicron, Alpha, Beta, Kappa, Gamma, SARS-CoV, and HCoV-NL63.

## Supplementary information

supplementary

## Data Availability

All data are available from the corresponding author on reasonable request.
