# Supplementary material for: ACE2-Targeting antibody suppresses SARS-CoV-2 Omicron and Delta variants
Source: Signal Transduct Target Ther. 2022 Feb 9;7:43. doi: 10.1038/s41392-022-00913-3 (PMC8828746; doi:10.1038/s41392-022-00913-3)
Supplement: Supplementary file 1 — supplementary [file 41392_2022_913_MOESM1_ESM.docx]

Supplementary Materials for

**ACE2-Targeting Antibody Suppresses SARS-CoV-2 Omicron and Delta Variants**

Jianxia Ou^3,†^, Yanan Zhang^2,4,†^, Yongmei Wang^1,4,†^, Zherui Zhang^2,4^, Hongping Wei^2,4^, Junping Yu^2,4^, Qi Wang^1,4^, Guifeng Wang^1,4,*^, Bo Zhang^2,4,*^, Chunhe Wang^1,4,*^

**This PDF file includes:**

Materials and Methods

**Materials and Methods**

Virus and cells

The SARS-CoV-2 Delta variant (IVCAS 6.7593) was originally isolated from a COVID-19 patient. The virus was amplified and titrated by a standard plaque forming assay on Vero E6 cells. Vero-E6 cells were cultured in Dulbecco’s modified Eagle’s medium (DMEM) containing 10% fetal bovine serum (FBS), 100 units/ml of penicillin and 100 µg/ml of streptomycin and maintained in 5% CO_2_ at 37°C.

Protein expression and purification

The His-tagged S1 proteins of Delta, Omicron, Kappa and ACE2-AVI biotinylated protein were purchased from Sino Biological (Beijing, China), and SARS-CoV-2 was purchased from ABclonal. ACE2-His, ACE2-Fc and 3E8 were prepared as previously described^1^. Purified plasmids were transfected into HEK293F cells (Shanghai Cell Line Bank, China) by polyethylenimine (Polysciences, Warrington, PA). Cells were then cultured in suspension in CD medium. After 5 days of culture, the supernatant was collected and purified by Ni-NTA or protein A chromatography. Size exclusion chromatography column (SEC) was used to examine the purity of the proteins.

Binding ELISA

96-well Immuno-plates (Greiner) were coated with 2.0 µg/ml of purified recombinant Fc-tagged human ACE2 protein at 4℃ overnight. After blocking at room temperature for 1 h with 1% casein (Thermo Fisher), plates were washed with PBS containing 0.05% Tween-20, and serial dilutions of 3E8 were added for 1 h incubation. After washing, goat anti-human Kappa conjugated with HRP (SouthernBiotech, 1:2000 dilution) was added and incubated for 1 h. Then, TMB substrate (Thermo Fisher Scientific) and 2 M of H_2_SO_4_ were added, and the OD_450_ was detected with a SpectraMax M5e (Molecular Devices) microplate reader. To measure the binding affinity of S1 proteins to ACE2, 2 μg/ml of various 6×His-tagged S1 proteins were coated onto plates followed by the addition of gradient diluted ACE2-Fc, and goat anti-human IgG conjugated with HRP was used for detection.

Neutralization ELISA

6×His tagged S1 proteins (2 μg/ml) were coated onto plates at 4 °C overnight. Serial dilutions of 3E8 or isotype were preincubated with 10 μg/ml of ACE2-Fc for 30 min at room temperature. Then, the mixture was added into the coated plate wells and incubated for 1 h. The bound ACE2-Fc was detected by HRP-conjugated goat anti-human IgG and developing substrate.

Biolayer interferometry (BLI)

3E8 with ACE2 binding affinities were measured by BLI using Fortebio Octet Red 96. For affinity measurement, 10 μg/ml of 3E8 was captured by a protein A biosensor to a level of 1.0 nm and incubated with different concentrations of hACE2-His protein. The baseline was established by PBS with 0.05% Tween-20 for 60 s. The association was set at 240 s, and the dissociation periods were set at 300 s. The mean Kon, Koff, and apparent KD values of binding affinities were calculated from all binding curves based on their global fit to 1:1.

For the affinity of S1-subunits from WT, Kappa and Delta SARS-CoV-2 to hACE2, 20 μg/ml of human ACE2-AVI biotinylated protein was immobilized by a SA biosensor (Sartorius) to a response level of 0.5 nm. Different concentrations of His-tagged S1 were applied in a two-fold dilution series in kinetics buffer. The association was set at 80 s, and the dissociation periods were set at 80 s. Data were reference subtracted and aligned to disassociation in ForteBio Data Analysis software 9.0. Data were calculated based on global fit to 1:1.

Flow Cytometry

Binding of S1-subunits from WT, Delta and Kappa SARS-CoV-2 with ACE2 on the cell surface was analyzed by flow cytometry using HEK293/ACE2/EGFP and Vero E6 cells. Cells were harvested and aliquoted into FACS tubes at 1×10^5^ cells/tube, washed with cold staining buffer (PBS+1% FBS), and then resuspended in 100 μl of different biotinylated S1 or 3E8 at different concentrations. The cells were kept at 4°C for 1 h on a shaker before being washed twice. The cells were resuspended in 100 μl of staining buffer containing APC-strepavidin (Biolegend) in the dark for 30 min, washed twice and resuspended in 200 μl of staining buffer for flow cytometry analysis.

For 3E8 blocking different S1 proteins binding to ACE2 on the cell surface, serial dilutions of 3E8 were incubated into a 96-well plate seeded with HEK293/ACE2/EGFP cells (1 × 10^5^ cells/well) at 4 °C for 1 h. Equal volumes of different biotinylated S1 proteins were mixed with the diluted antibodies to a final concentration of 2 μg/ml. Following 60 min of incubation at 4°C, the cells were washed and incubated with APC-Streptavidin (Biolegend) for another 30 min. The mean fluorescence intensity (MFI) for S1 protein binding to the cell surface was set as 100%. Blocking (%) = [1−(MFI_samples_−MFI_unstain_)/(MFI _positive control_−MFI_unstain_)] ×100%.

Pseudo-typed virus neutralization assay

Pseudo-typed WT, Delta, Omicron and Kappa SARS-CoV-2 were constructed by co-transfection of two plasmids, one expressing Env-defective HIV-1 with a luciferase reporter (pNL4-3.luc. RE) and the other expressing the full-length S-protein of WT, Delta and Kappa SARS-CoV-2 into HEK293T cells. The supernatant containing virus particles was harvested 48 h post-transfection followed by 0.45 μm filtration. HEK293F/ACE2/EGFP cells were pre-seeded with 1.2 × 10^4^ cells per well in a 96-well plate. The confluent cells were incubated with 50 μl of serially diluted antibodies or ACE2-Fc for 1 h at 37°C followed by the addition of various pseudoviruses of the same volume. In all, 100 μl of DMEM with 10% FBS was added as a negative control. In total, 100 μl of pseudovirus and DMEM mixed at a ratio of 1:1 was used as a positive control. After 24 h, the medium was changed, and the cells were incubated for another 48 h. The relative light units (RLUs) of luminescence were measured by a Firefly Luciferase Reporter Assay Kit (Meilunbio). Neutralization (%) = [1−(RLU_samples_−RLU_negtive control_)/(RLU_positive control_−RLU_negtive control_)] ×100%. The *IC_50_* values were calculated by non-linear.

COVID-19 mouse model and disease suppression

TgTn (CAG-human ACE2-IRES-Luciferase -WPRE-polyA) mice were purchased from Shanghai MODEL organisms and cared in accordance with the recommendations of the National Institutes of Health Guidelines for the Care and Use of Experimental Animals. All animal studies were conducted in a biosafety level 3 (BSL-3) facility at Wuhan Institute of Virology under a protocol approved by the Laboratory Animal Ethics Committee of Wuhan Institute of Virology, Chinese Academy of Sciences (Permit number: WIVA26201701).

Four groups of eight- to ten-week-old female TgTn mice (*n* = 5 per group) were treated with 3E8, ACE2-Ig or isotype monoclonal antibody at a concentration of 10 mg/kg by the intraperitoneal route. Twelve hours later, all mice were intranasally infected with 10^5^ PFU Delta variant in a total volume of 50 μl. The mice were monitored for body weight changes during three days after infection, and at 3 days post-infection (dpi), the lungs of mice were collected for viral titer determination by plaque assay, fixed with 4% paraformaldehyde, embedded in paraffin, sagittally sectioned at 4-μm thickness on a *microtome*, and mounted on APS-coated slides for H&E staining.

Plaque assay

A total of 10^5^ Vero-E6 cells per well were seeded into 24-well plates one day before the plaque assay. A series of 1:10 dilutions were made by mixing 15 μl of virus sample with 135 μl of DMEM. Then, 100 μl of each dilution was added to individual wells of 24-well plates containing confluent Vero-E6 cells. The plates were incubated at 37°C with 5% CO_2_ for 1 h before a layer of 2% methyl cellulose was added. After 4 days of incubation at 37°C with 5% CO_2_, the cells were fixed with 3.7% formaldehyde and then stained with 1% crystal violet. Plaque morphology and numbers were recorded after washing the plates with tap water.

**References and Notes**

1. Chen, Y. *et al.* ACE2-targeting monoclonal antibody as potent and broad-spectrum coronavirus blocker. *Signal transduction and targeted therapy* **6**, 315 (2021).
